# Patterns of Diversity, Areas of Endemism, and Multiple Glacial Refuges for Freshwater Crabs of the Genus *Sinopotamon* in China (Decapoda: Brachyura: Potamidae)

**DOI:** 10.1371/journal.pone.0053143

**Published:** 2013-01-04

**Authors:** Fang Fang, Hongying Sun, Qiang Zhao, Congtian Lin, Yufang Sun, Wei Gao, Juanjuan Xu, Junying Zhou, Feng Ge, Naifa Liu

**Affiliations:** 1 Jiangsu Key Laboratory for Biodiversity and Biotechnology, College of Life Sciences, Nanjing Normal University, Nanjing, China; 2 College of Life Sciences, Nanjing Normal University, Nanjing, China; 3 Key Laboratory of Animal Ecology and Conservation Biology, Institute of Zoology, Chinese Academy of Sciences, Beijing, China; 4 Nanjing Institute of Environmental Sciences, Nanjing, China; 5 School of Life Sciences, Lanzou University, Lanzhou, China; Lund University, Sweden

## Abstract

Previous research has shown that the geographical distribution patterns of freshwater fishes and amphibians have been influenced by past climatic oscillations in China resulting from Pleistocene glacial activity. However, it remains unknown how these past changes have impacted the present-day distribution of Chinese freshwater crabs. This work describes the diversity and endemism of freshwater crabs belonging to *Sinopotamon*, a highly speciose genus endemic to China, and evaluates its distribution in terms of topography and past climatic fluctuations. Species diversity within *Sinopotamon* was found to be concentrated in an area from the northeastern edge of the Yunnan-Guizhou Plateau to the Jiangnan Hills, and three areas of endemism were identified. Multiple regression analysis between current climatic variables and *Sinopotamon* diversity suggested that regional annual precipitation, minimum temperature in the coldest month, and annual temperature range significantly influenced species diversity and may explain the diversity patterns of *Sinopotamon*. A comparison of ecological niche models (ENMs) between current conditions and the last glacial maximum (LGM) showed that suitable habitat for *Sinopotamon* in China severely contracted during the LGM. The coincidence of ENMs and the areas of endemism indicated that southeast of the Daba Mountains, and central and southeastern China, are potential Pleistocene refuges for *Sinopotamon*. The presence of multiple Pleistocene refuges within the range of this genus could further promote inter- and intraspecific differentiations, and may have led to high *Sinopotamon* species diversity, a high endemism rate and widespread distribution.

## Introduction

Inland water and freshwater biodiversity are invaluable natural resources, yet freshwater habitats and the species they support are among the most threatened ecosystems worldwide [1], [2]. Freshwater crabs play key ecological roles because of their relatively high abundance and biomass [3], [4], [5] and position in food webs as primary and secondary consumers [6], [7]. Recent assessments suggest that 32% of global freshwater crabs are threatened [8]. China is one of the most species-rich areas for true freshwater crabs in the world, however, assessments of threatened freshwater crabs are seriously lacking and data deficient [8], [9].

**Figure 1:**
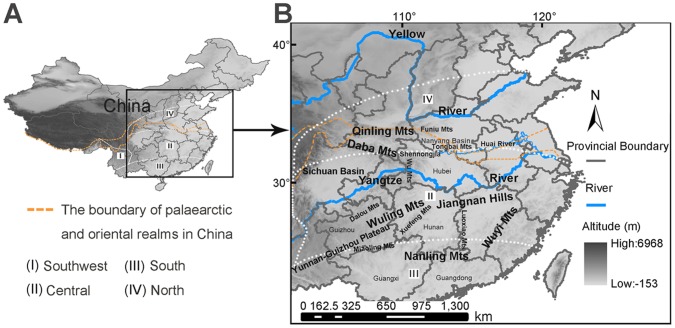
The study area and topography. A. The study area is squared in the map of China. Hypothesized zoogeographical regions (Southwest, Central, South and North regions) for Chinese freshwater crabs are separated by white dashed lines. Boundaries of the Palaearctic and Oriental realms are orange dashed lines. **B.** Topographic map of central-eastern China showing major mountain ranges, plateaus, basins and rivers. All geographical features quoted in the text are indicated.

**Figure 2:**
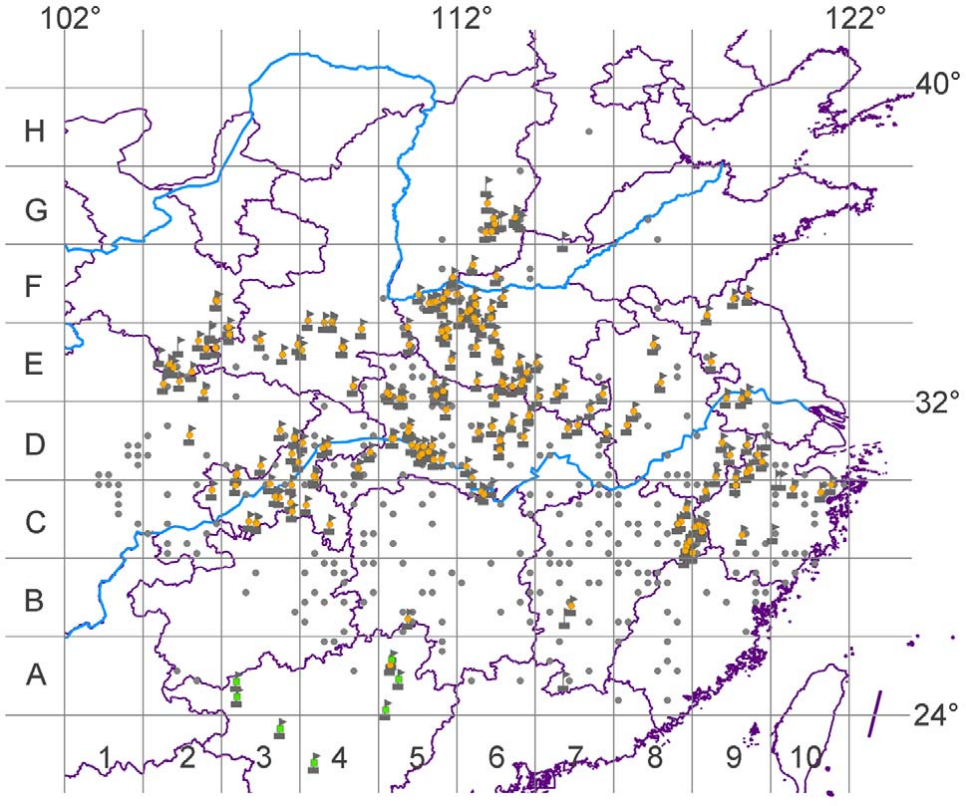
The distribution of *Sinopotamon* spp. endemic to mainland China. Grey banners are our field investigation locations. We obtained *Sinopotamon* specimens from the locations indicated by orange colour dots. Grey dots represent historical distribution data. Green quadrates are freshwater crabs belonging to other genera.

Freshwater crabs in the genus *Sinopotamon* Bott, 1967 are among the most striking freshwater benthic macro-invertebrates endemic to China. Since the first description by Milne-Edwards in 1853 of *Sinopotamon* spp. in the middle reaches of the Yangtze River [10], [11], many species have been described across continental China. Driven by investigations into parasitic metacercariae of flukes in local freshwater crabs [12], by 1999, 70 species (and five subspecies) were recognized in the genus [10]. The lists of freshwater crabs across Asian and China are regularly updated [8], [9], [13], [14], and at present 80 species (and four subspecies) are included in the *Sinopotamon*, making it the most species-rich freshwater crab genus in Asia [14]. *Sinopotamon* also has the largest geographic distribution of freshwater crabs in China, inhabiting three of the four geographical regions hosting freshwater crabs in China [10] (Figs. 1a, b). For example, the Oriental and Palaearctic zones, two widely accepted zoogeographical realms dividing China [15], [16], [17] Contain *Sinopotamon* species, whereas almost all other freshwater crabs inhabit the Oriental zone only (Figs. 1b, 2). As primarily amphibious organisms with direct development, maternal care and specific habitat requirements [10], freshwater crabs have limited dispersal, local-scale distributions, and are heavily influenced by topography and climatic fluctuation. Why *Sinopotamon* includes such a high number of species and is so widespread remains unclear. Addressing this problem will provide a better understanding of the distribution patterns of *Sinopotamon* and enhance our knowledge of biogeography and conservation-based assessments for freshwater crabs in China.

The vast regions occupied by *Sinopotamon* species (Fig. 1b) are located east of the Qinling Mountains, the middle and lower reaches of the Yellow and Yangtze Rivers, and to northern Nanling Mounains. These areas have experienced profound climatic changes since the Pleistocene: the formation of the East Asian monsoon, climatic fluctuations during glacial cooling and interglacial warming, differentiation of environments following uplifting of the Qinghai-Tibet Plateau, and accompanying changes in river systems [18], [19], [20], [21]. Recent studies show that complex topography and mountain networks provided relatively stable micro-ecosystems as potential suitable habitat for temperate species during glacial periods [22]. Multiple refuges for various plants and vertebrates are located in mountainous areas in central, eastern, and southern China, providing suitable habitat for survival during Pleistocene glaciations [23], [24], [25], [26], [27], [28], [29], [30], [31], [32]. However, it remains unknown how *Sinopotamon* spp. responded to the regional tectonic and climatic changes since the Pleistocene.

Here we identified diversity patterns and areas of endemism of *Sinopotamon*, and evaluated its distribution in terms of regional topography and past climatic fluctuation. Many studies have suggested that multivariate methods can reveal informative biogeographical patterns [33], [34], [35], because one can reduce the inherent complexity of biogeographical data and obtain replicable results [36]. In this study, we analyzed diversity patterns of *Sinopotamon* using geographical information systems (GIS) and identified areas of endemism (AOEs) using a combination of parsimony analysis endemicity (PAE) and clustering analysis. Methodologies such as PAE and clustering complement each other in identifying AOEs. We also incorporated ecological niche models (ENMs) based on palaeoclimatic and current climatic data to infer the potential historical distribution patterns of species, in turn helping to assess the impact of glaciations and explain current distribution patterns.

The aims of this study were to (1) describe the diversity and endemism of *Sinopotamon* species; (2) indentify dominant climate factors explaining the diversity patterns of *Sinopotamon* species; and (3) assess the impact of Pleistocene glaciations on the distribution of *Sinopotamon*.

## Methods

### Ethics Statement

This study was conducted according to laws on animal welfare and research in China, and was approved by the Animal Ethics and Welfare Committee of Zoological Society of Jiangsu Province (Permit No. AREWC 20907).

### Study Area

The study area is located 24–40°N and 102–122°E and includes central eastern China across drainages of the Yellow and Yangtze Rivers (Figs. 1a & 1b).

### Establishing the Distribution Database

Except one species (*S. koatenense*) that was not used for our analysis because of an ambiguous location record in western Fujian province, a database comprising species names for the remaining 79 (and four subspecies) *Sinopotamon* species and their distribution sites was compiled based on long-term field surveys, sample collection from October 2000 to September 2011 (Fig. 2), and all known published assessments of this genus [10], [12], [13], [37], [38], [39], [40], [41], [42], [43], [44], [45], [46], [47], [48], [49], [50]. This database contained 638 *Sinopotamon* specimen locations including 253 investigation sites was imported into ArcGIS v9.3 (ESRI, Redlands, USA) for mapping.

### GIS Analysis

Geographical information systems can map distributions of taxa and detect centres of species diversity or endemism [51], [52], [53], [54]. To highlight diversity centres, a density map for *Sinopotamon* spp. per 2°cell was created based on the 638 specimen locations by overlaying the distributions of species within the genus. Species diversity (number of species in individual cells) was then shown statistically [55].

### Parsimony Analysis of Endemicity

Areas of endemism are defined as areas delimited by the congruent distribution of two or more species of restricted range [56], [57], [58]. These areas are valuable when defining primary biogeographical homologies and investigating relationships between areas in historical biogeography [59], [60], [61], [62], and assist in the identification of priorities for biodiversity conservation [63], [64]. PAE is a biogeographical tool that aims to classify areas by the most parsimonious solution based on the shared presence of taxa [65], [66], [67]. Although there is a continued debate about the role of PAE [66], [68], [69], it has proven to be a useful and important tool for identifying AOEs [58], [70], [71], [72]. We used PAE to identify areas of endemism following Morrone [54]. Some researchers have advised using taxa at multiple taxonomic levels in PAE [73], [74] and we used the distributional data of both species and subspecies. Based on data from 83 *Sinopotamon* taxa (including four subspecies), two grids of operative geographical units (OGUs) were constructed using two different scales, 1°×1° and 2°×2°. We then performed a preliminary analysis of the suitability of each group of OGUs because PAE can be sensitive to the scale used [71]. On the basis of this analysis we chose the 2°×2° grid to avoid fragmentation of the distributional data. Fifty-two OGUs contained records of endemic *Sinopotamon* species (Fig. 2); among these, 40 OGUs containing two or more endemic *Sinopotamon* species were used in the final PAE analysis. OGUs in which only one species was present or with no recorded taxa were excluded from the PAE because they are not informative. Forty-two taxa that had precise distributional sites were used in the final PAE.

A matrix of 41 OGUs×42 taxa was constructed. A hypothetical outgroup OGU with ‘0′ for all columns was added to root the trees [58], [71]. PAE was implemented using PAUP 4.0 [75]. A 50% majority consensus tree of equally parsimonious trees from the PAE analysis was obtained. We evaluated relative support for each branch using bootstrapping with 1000 replicates and tree bisection-reconnection (TBR) swapping to estimate the validity of the OGU groups [76]. The frequency of occurrence of bipartitions for the majority consensus tree was estimated. Branches with relatively high bootstrap values (>50%) were chosen as candidates for areas of endemism.

### Clustering

Clustering is different from the parsimony analysis of endemicity in that it is based on whole-assemblage similarities [34]. The central aim of clustering is to classify similar objects into respective groups [34]. Based on a data matrix of 52 OGUs×83 taxa, an unweighted pair-group method using arithmetic averages (UPGMA) with Jaccard similarity was calculated and plotted [77], [78]. This technique provides an unweighted arithmetic average between each individual object and other members of the cluster or between members of clusters as they merge [79]. The validity of clustering results was evaluated using the co-phenetic correlation coefficient [80], [81], which correlates pairwise distance from the leaves of a dendrogram to the encompassing node with the distances in the original distance matrix. It thus represents a direct measure of how much of the original information is retained in the dendrogram [80]. All procedures were performed using R [82].

### Maxent Analysis

On the basis of current climatic conditions and those of the last glacial maximum (LGM), we constructed ENMs for *Sinopotamon* species using Maxent v3.2.1 [83], [84]. Maxent determines a probability distribution of maximum entropy by combining presence-only data with ecological layers. This method has been used in several recent studies comparing current and historical species distributions [85], [86], [87] and performs well compared to other methods in predicting current and past species distribution range [88], [89]. Here, potential suitable habitat was predicted respectively for the nine single species based on enough recorded data of each species that is fit for ENMs (e.g. 70 sites for *S. acutum*, 65 sites for *S. yangtsekiense*, 53 sites for *S. honanense*, 38 sites for *S. lansi*, 30 sites for *S. shensiense*, 29 sites for *S. chekiangense*, 25 sites for *S. davidi* and *S. depressum* respectively, and 24 sites for *S. fukienense*). Given the limitation of nine single species models in determining the distribution of the genus *Sinopotamon*, the 638 sites for 79 species were combined for ENMs. Niche models are vulnerable to sample size and the geographical distribution of records [90], and duplicate presence can result in pseudoreplicated niche models [91]. In order to minimize such effects we eliminated data sites within *Sinopotamon* following the method of Debandi et al. [92]. We eliminated all duplicate occurrences and ran 10 models to obtain basic statistics of the performance. Subsequently, we eliminated localities that were within a distance range of 5, 10 and 25 km of each other, maintaining the best georeferenced data sites, and running 10 models each time. Distance among data sites was calculated using Geographic Distance Matrix Generator v1.2.3 (http://biodiversityinformatics.amnh.org/open_source/gdmg/index. php). We used the default parameters of Maxent (convergence threshold (10^−5^), 10000 background points, maximum number of iterations (500), a regularization multiplier of 1 and autofeatures) with the logistic probabilities used for output [84]. Model accuracy was evaluated by assessing the area under the receiver operating characteristic (ROC) curve (AUC) [83]: a threshold-independent index that ranges from 0.5 (randomness) to 1(exact match), whereby scores higher than 0.7 indicate good model performance [93]. Seventy-five percent of the presence data were randomly selected by Maxent to train the model and the remaining 25% to test model performance. We converted the logistic probability values to presence-absence data using the ‘minimum training presence’ threshold, where the omission rate is set to zero. The database containing localities within a range of 5 km provided good balance between higher AUC training and test value, lower AUC difference, and a suitable number of data sites, and was therefore used in analyses. Thus, the original database of the genus was reduced to 455 sites of 76 species. All predictions were mapped in ArcGIS v9.3 (ESRI, Redlands, USA).

Nineteen bioclimatic variables were downloaded from the WorldClim database [94] for current conditions and for the LGM at 2.5 min spatial resolution (c. 5 km). LGM data were obtained from two different general circulation model (GCM) simulations: the Community Climate System Model (CCSM) [95] and the Model for Interdisciplinary Research on Climate (MIROC) [96]. Environmental layers for each model were selected based on preliminary work identifying the most informative bioclimatic predictors. Correlation analysis within the 19 environmental variables was calculated to reduce redundant information, and those variables with a correlation index higher than 0.9 were eliminated. After that, we discarded variables if they failed to provide any significant contribution to the predictive model. The environmental variables being used to construct models for the nine single species and the 76 *Sinopotamon* species are shown in Table S1. The variables are derived from monthly temperature and precipitation data layers, and represent biologically meaningful aspects of climate variation [86], [87].

In order to obtain a robust model result and given differences between the two models’ outputs (CCSM and MIROC) for the LGM, we combined datasets and obtained a consensus. We followed the conservative methods of Waltari et al. [87] and Flanders et al. [23] and showed areas predicted as suitable by both models and discarded areas where no agreement occurred.

### Statistical Analyses

To look for relationships between *Sinopotamon* species diversity and climatic factors, we selected 10 bioclimatic variables (annual mean temperature, temperature seasonality, minimum temperature in the coldest month, annual temperature range, mean temperature in the coldest quarter, annual precipitation, precipitation in the driest month, precipitation seasonality, precipitation in the driest quarter and precipitation in the coldest quarter) relevant to the habitat models of *Sinopotamon* species. The selected variables also behave both as contributors under the Maxent species distribution model and have a high magnitude of regularized training gain (RTG) on the Jackknife test of variable importance (RTG >0.78; [84]). We obtained the extractions of climatic variables and species diversity at 2° cells through GIS. Statistical analyses were carried out using the Car package [97] for the statistical program R [82]. A Kolmogorov-Smirnov normality test were performed in R [98], [99]. In order to control the effects of collinearity on the estimation of the regression coefficients, a collinearity analysis [100], [101] was conducted based on the ten climatic variables. Multiple linear regression analysis [102], [103] was performed to confirm the dominant factors accounting for patterns of *Sinopotamon* species diversity.

## Results

### Diversity Patterns

**Figure 3:**
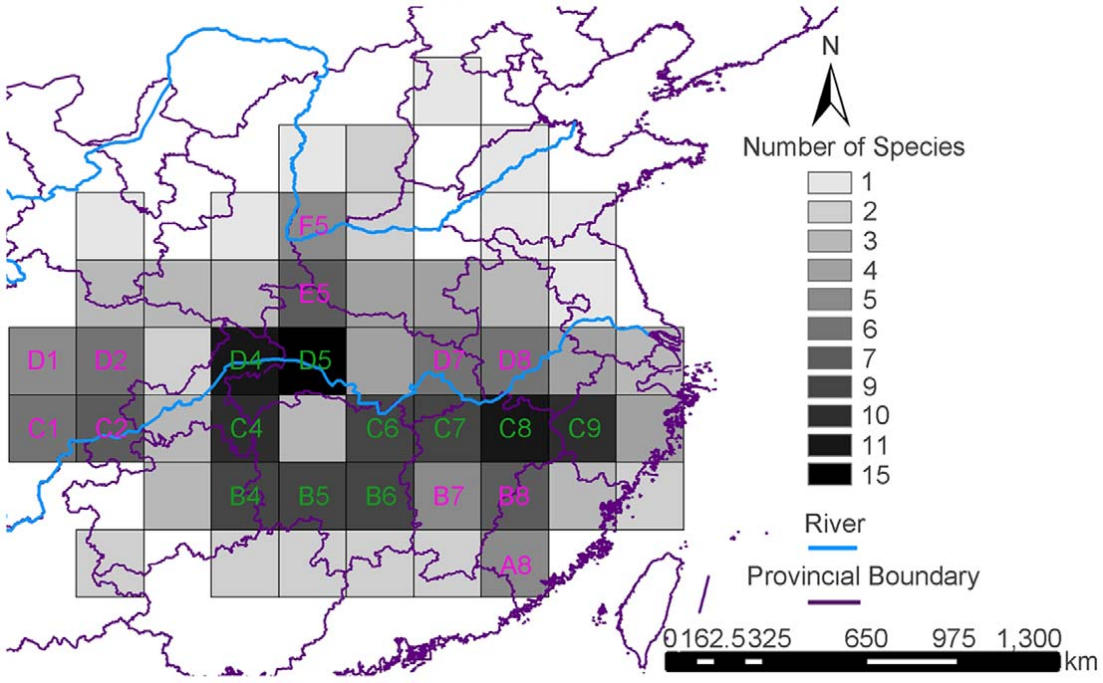
Map of species diversity of *Sinopotamon* spp. in China per 2°cell. OGUs with more than 4 species were indicated by corresponding labels.

*Sinopotamon* species inhabit mountains, hills and plains across large basins formed by the Yellow and Yangtze Rivers (24.3–38.9°N, 102.8–121.9°E; Fig. 2). Figure 3 shows that *Sinopotamon* species diversity is concentrated from the northeastern edge of the Yunnan-Guizhou Plateau to the Jiangnan Hills (B4–6, C4, C6–9, D4–5 in Fig. 3; Table S2), where 58 *Sinopotamon* species are found, and hence represents the distribution centre. The northeastern edge of the Yunnan-Guizhou Plateau includes Wushan, eastern Daba Mountains (see D4–5 in Fig. 3), Wuling Mountains (see B4 and C4 in Fig. 3) and the Xuefeng Mountains (see B5 in Fig. 3). These mountainous areas are usually above 1000–1500 m sea level (a.s.l.) and located in the eastern margin of the second step and turning point of the terrain in mainland China [104]. The Jiangnan Hills are located east of the Xuefeng Mountains to the northeastern section of the Wuyi Mountains, and from north of the Nanling Mountains to south of the Yangtze River (see B6, C6–9 in Fig. 3). These regions include many hills and mountains (500–1000 m a.s.l.) in a northeast-southwest direction [104]. Sichuan Basin and the areas adjacent to the abundant-centre harbored 40 *Sinopotamon* species (A8, B7–8, C1–2, D1–2, D7–8, E5, F5; Fig. 3; Table S2). However, the lowest species diversity is symmetrically distributed in the northernmost and southernmost marginal regions across the whole distribution range (each region embraces seven species; Fig. 3; Table S2).

### Areas of Endemism

**Figure 4:**
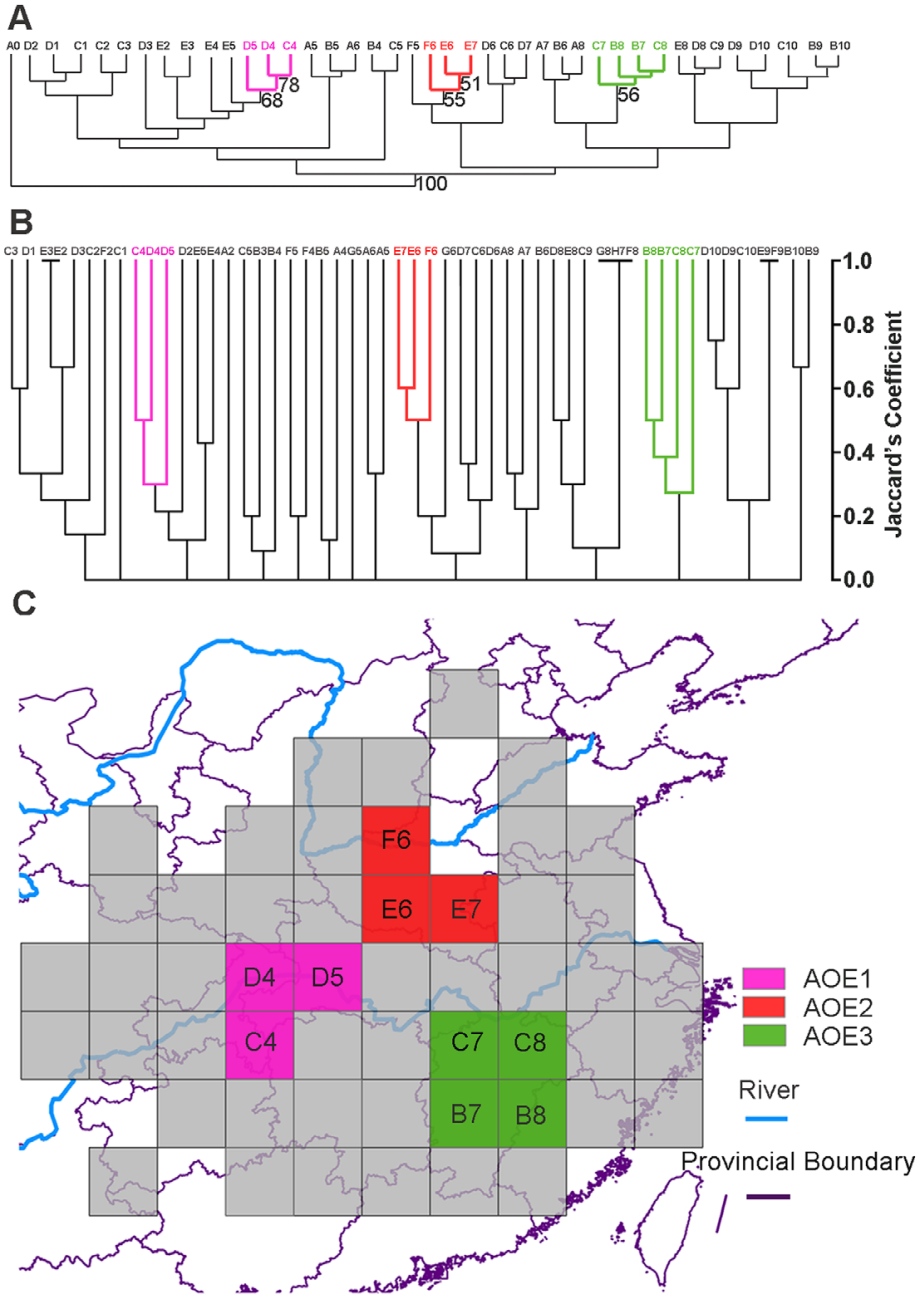
Areas of endemism inferred from PAE and clustering. A. OGUs (quadrates) were obtained based on 2 degree grid. Fifty % majority consensus tree obtained from the PAE analysis. The terminals correspond to the OGUs shown in Fig. 2. A0 is the out group. Bootstrap values (above the branches) for relatively highly supported branches are shown. **B.** UPGMA tree of 83 species and subspecies and fifty-two OGUs based on Jaccard’s similarity coefficient. Cophenetic correlation was 0.8066. **C.** Three AOEs were identified by PAE and clustering: AOE1- southeast of Daba Mountains, AOE2- eastern Funiu Mountain, AOE3- the Jiangnan Hills.

For all taxa examined, 42 species were parsimony-informative, and 90 most-parsimonious trees were obtained. The 50% majority consensus tree (tree length = 102, CI = 0.441, RI = 0.612) is shown in Figure 4a. Bootstrap values with relatively highly supported branches (>50) are shown. The phenogram from UPGMA (Fig. 4b) showed very similar patterns with the phylogram from the PAE (Fig. 4a). The Cophenetic correlation in UPGMA was 0.8066. Under the criterion that areas of endemism contain at least two species restricted to a group of OUGs, three areas of endemism (see Fig. 4c) were identified and are supported by UPGMA and PAE as follows: (1) southeast of Daba Mountains including the northwestern Wuling Mountains, Wushan and Shennongjia (AOE1); (2) eastern Funiu Mountains includes areas from the eastern part of the Qinling Mountains to northern sections of the Tongbai Mountains (AOE2); and (3) the Jiangnan Hills comprising an area extending from the eastern Luoxiao Mountains to northwestern Wuyi Mountains (AOE3). The endemic species used to delimit the AOEs (species that are synapomorphic for each group of OGUs) are listed in Table S3.

### Model Prediction of Geographical Distribution of *Sinopotamon*

**Figure 5:**
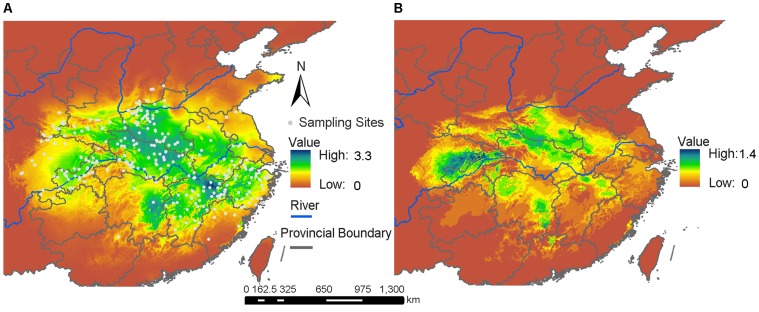
Overlapping ecological niche models (ENMs) of nine *Sinopotamon* species. Output shows marginal (green) to optimal (dark blue) habitat. Sampling locations of nine species used to build ENMs shown as filled circles. **A.** Overlapping ENMs for current condition. **B.** Overlapping ENMs for the Last Glacial Maximum.

**Figure 6:**
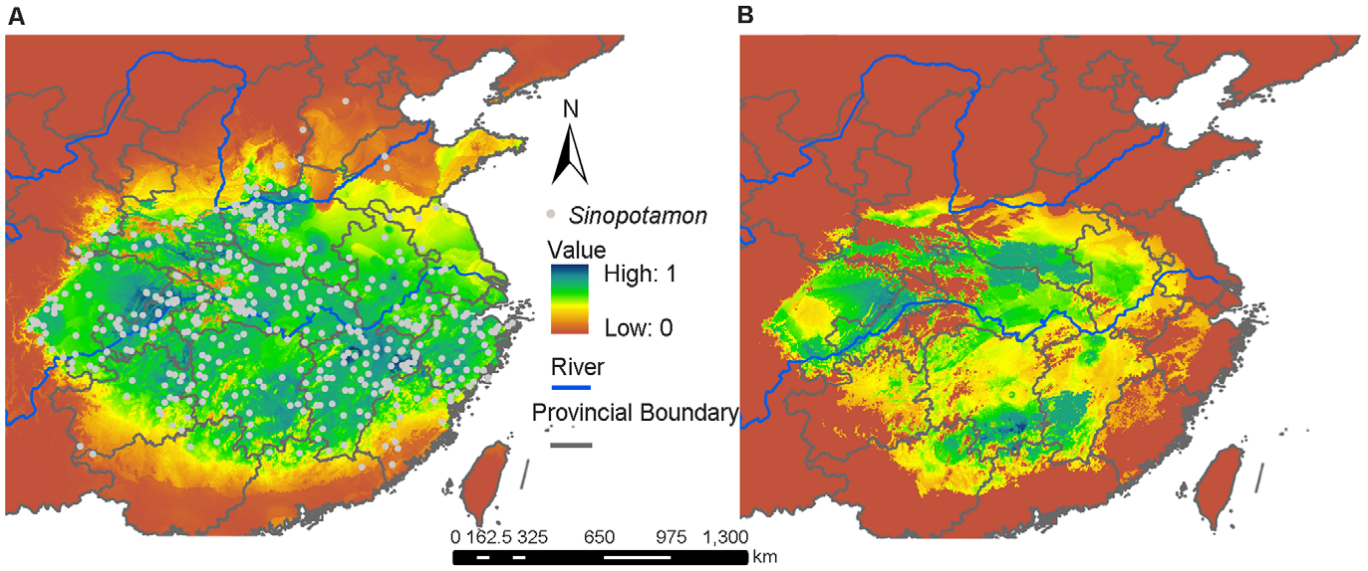
Predicted *Sinopotamon* spp. distributions from ecological niche models using Maxent. Output shows marginal (green) to optimal (dark blue) habitat. Sampling locations used to build ENMs shown as filled circles. **A.** ENMs for current condition. **B.** ENMs for the Last Glacial Maximum.

For predicting the representative suitable habitat of the genus *Sinopotamon*, we built ENMs for the nine species, *S. acutum*, *S. davidi*, *S. depressum*, *S. honanense*, *S. shensiense*, *S. yangtsekiense*, *S. lansi*, *S. chekiangense* and *S. fukienense* (Figs. S1, S2, S3). Each ten replicate runs obtained the same AUC values. The AUC for training data and test data had values higher than 0.95 in all analyses, which implied that the results greatly differed from random prediction. We used GIS to superimpose the modeling layers of each species and created a combined map in current and LGM periods as shown in Figure 5a & b. The result suggested that the suitable habitats of these species contracted heavily in their ranges during the LGM (Figs. 5a & b), and were concentrated in three main regions. First: southeastern Daba Mountains (Figs. 5b, S1b) harboring three species populations, *S. acutum*, *S. davidi*, and *S. depressum*. Second: southeastern Qinling Mountains including low areas between the Qinling Mountains and Daba Mountains, northeastern Funiu Mountains, the Nanyang basin and southeast of the Tongbai Mountains (Figs. 5b, S2b), providing appropriate conditions for three species populations, *S. shensiense*, *S. honanense* and *S. yangtsekiense*, a partial population of the *S. yangtsekiense* also contracted southeastward to the Jiannan Hills in northern Wuyi Mountains (Fig. S2b). But, the suitable areas for each species were not overlapped fully. Third: geographically separated into several fragments around the Jiannan Hills, northwest of the Wuyi Mountains, north of the Nanling Mountains, and a small region west of the Luoxiao Mountains (Figs. 5b, S3b), where the remaining three species (*S. lansi*, *S. chekiangense* and *S. fukienense*) inhabited during the LGM. The geographical distribution of the whole *Sinopotamon* was well modeled. The current distribution range that we predicted closely matches that observed (AUC = 0.944 for training data and AUC = 0.936 for test data) (Fig. 6a). As we predicted suitable habitat during the LGM was severely contracted in range and concentrated in three separated regions (southeast of Daba Mountains, the southeast section of the Qinling Mountains, and north of the Nanling Mountains and the western Wuyi Mountains) (Fig. 6b). These results were largely consistent with the three main regions modeled from the nine focal species during the LGM.

### Multiple Linear Regression Analysis

The Kolmogorov-Smirnov normality test showed that the distribution of the climatic data was normal (Kolmogorov-Smirnov test, *p*>0.05). The collinearity analysis found that annual mean temperature, temperature seasonality and mean temperature in the coldest quarter are collinear. Then, the seven non-collinear variables, coupled with the largest coefficient variable of those three collinear variables were selected for multiple linear regression analysis, with the logarithm of species diversity as the dependent variable. By multiple linear regression analysis, after adjustment for cofactors, the relationship between species diversity and climate variables was determined using a linear multiple regression model, which provided a significant fit for species diversity (*F*_8,43_ = 4.592, *p* = 0.001, adjusted *R^2^* = 0.360; Table 1). Species diversity was predicted positively associated with annual precipitation (*t* = 2.396, *p*<0.05) and minimum temperature in coldest month (*t* = 2.304, *p*<0.05), but negatively associated with annual temperature range (*t* = −2.798, *p*<0.01) (Table 1).

**Table 1:** Multiple regression analysis between logarithm of species diversity and climate variables in current condition.

	Estimate	Std. Error	t value	Pr (>|t|)
Intercept	−4.762	6.354	−0.749	0.458
BIO1	−0.014	0.176	−0.078	0.938
BIO4	0.761	1.009	0.754	0.455
BIO6	0.155	0.067	2.304	0.026*
BIO7	−2.081	0.744	−2.798	0.007 **
BIO11	0.077	0.068	1.143	0.259
BIO12	1.336	0.558	2.396	0.021*
BIO14	0.898	0.713	1.259	0.215
BIO19	−0.606	0.780	−0.777	0.441

Signif. codes: ** *p*<0.01, * *p*<0.05; Residual standard error: 0.615 on 43 degrees of freedom; Multiple R-squared: 0.461, Adjusted R-squared: 0.360; F-statistic: 4.592 on 8 and 43 DF, p-value: 0.001.

BIO1-Annual Mean Temperature, BIO4-Temperature Seasonality, BIO6-Minimum Temperature in the Coldest Month, BIO7-Annual Temperature Range, BIO11-Mean Temperature in the Coldest Quarter, BIO12-Annual Precipitation, BIO14-Precipitation in the Driest Month, BIO19-Precipitation in the Coldest Quarter.

## Discussion

### Climate Factors as Drivers of Distribution Patterns of Species Diversity

The modern geographic distribution of *Sinopotamon* spans temperate to subtropical regions. The phased uplift of the Qinghai-Tibet Plateau led to high western and low eastern terrain and creation of large mountains following a west-east orientation. The second and the third steps (less than 2000 m a.s.l.) have subsequently characterized the topography of central eastern China [105]. These complex geological events probably triggered climate change in these regions, which in turn led to biotic reorganization [106]. *Sinopotamon* species require warm and moist climates for survival, reproduction, population growth, prolonged activity outside water, and migration into neighboring streams. As the Qinling and Daba Mountains could form a natural boundary between northwest and southeast China, central eastern China is characterized by a warm temperate to subtropical climate [107] where the *Sinopotamon* species are now widely found. Their species richness was proposed to be the highest in Hubei and Hunan provinces through analysis based on provincial units [14]. However, in our study more detailed high-density mapping in a two degree grid was done to obtain a finer resolution distribution pattern and yield correlations with environmental parameters [108]. Our results suggest that *Sinopotamon* diversity is concentrated in areas from the northeastern edge of the Yunnan-Guizhou Plateau to the Jiangnan Hills (Fig. 3). Multiple regression analysis based on current climate variables suggested that three climatic factors (regional annual precipitation, minimum temperature in the coldest month and annual temperature range) significantly influenced species diversity of the *Sinopotamon* and may be major factors driving diversity within this group. Freshwaters forms the basis of life for freshwater crabs and is usually affected by precipitation regimes [109]. The spatial pattern of precipitation in these areas is significantly influenced by terrain with more rainfall in mountains than in plains [110]. The regions, located at the geographical center of the genus’ range (from the northeast edge of the Yunnan-Guizhou Plateau to the Jiangnan Hills), receive over 1000 mm annual rainfall and range from 4 to 8°C in the coldest month [104]. The annual temperature range in this region is 22 to 26°C [104]. Combined, these are optimum conditions for *Sinopotamon*, leading to an extraordinary high number of *Sinopotamon* species (Fig. 3). Since the Qinling and Daba Mountains block cold air moving southward in winter, the Sichuan Basin (characterized by a subtropical monsoon and basin-terrain surrounded by high mountains) forms hot and humid climatic conditions (annual temperature range: 18 to 22°C; temperature in coldest month: 0 to 5°C; annual rainfall >800 mm; [104]) and exhibits a relative high species diversity (Fig. 3).

Areas located near the northernmost edge of the geographical range of *Sinopotamon* had the lowest species diversity. This may be because of the dry and cold climate (annual rainfall is less than 800 mm; temperature in the coldest month is −4 to 0°C; [104]) and extreme temperatures fluctuation (annual temperature range varied from 26 to 30°C; [104]) in this area. Since *Sinopotamon* are generally warmth and moisture-loving organism, their physiological intolerance for aridity, cooling and large temperature range could lead to the lowest species diversity in the northern boundary of their distribution range. Low *Sinopotamon* species diversity at the southern boundary of the distribution was also found, and may be the result of competition between species of related genera, despite a humid and warm climate in that area suitable for *Sinopotamon*. This idea is supported by the abundance of freshwater crabs at the genus and species level in southern provinces such as Guizhou (11 genera, 27 species), Guangxi (12 genera, 30 species) and Guangdong (six genera, 16 species) ([10]; Fig. 2).

### Areas of Endemism and Multiple Glaciation Refuges for the *Sinopotamon*

This study indentified three areas of endemism (AOE1, AOE2 and AOE3) for *Sinopotamon* based on the combination of PAE and clustering (Fig. 4). The three areas of endemism largely coincided with the three most suitable habitats as revealed by ENMS during LGM (Figs. 4 & 6b). The first was located in the southeastern Daba Mountains and embraced the highest species diversity; the second was located in central China including the southeastern Funiu Mountains, the Nanyang basin, and the Tongbai Mountains; the third was located in the southeast China from northeastern Nanling Mountains to north of the Wuyi Mountains and also exhibited relatively high species diversity. A close relationship between areas of endemism and glacial refugia is widely accepted [76], [111], [112], [113], and the distribution ranges of endemic species for plants are usually used to infer the location of refugia [51], [108], [114]. Furthermore, ecological niche models based on palaeo and current climate data and presence-only species records have been suggested as tools to understand the processes that have shaped current distribution patterns of species and infer potential refuges [23], [86], [87], [115]. The coincidence of areas of endemism and glacial model niches for *Sinopotamon* in this study supports the idea of refugia existing in three areas (Fig. 6b). Further predicted suitable habitat for the seven single species (*S. acutum*, *S. davidi*, *S. depressum*, *S. honanese*, *S. lansi*, *S. chekiangense* and *S. fukienense*) were contracted into either of the three refugia during the LGM, while contracted suitable habitat of *S. yangtsekiense* was separated into the central and southeast China refugia. The locations of areas of endemism are generally attributed to historical processes, such as the existence of past glacial refuges. Not all of the glacial relict species migrate out from their refugia and recolonize formerly glaciated or inhospitable areas following the retreat of glaciations [27]. It was likely that many refugial *Sinopotamon* species with weak dispersal ability and narrow environmental tolerances remained and were restricted to the refugial areas, resulting in three areas of endemism across the modern distribution range. Regions with many endemic taxa, obviously, should obtain high conservation priorities [64]. The areas of *Sinopotamon* endemism revealed in this study deserve more attention from conservation professionals.

The extent of the impact of Pleistocene climatic oscillations on the distribution of freshwater organisms in China remains an interesting issue. Researchers have shown that climatic oscillations have deeply impacted the distributions of freshwater fishes and amphibians [31], [32], [116], [117], [118], [119], [120]. We show that the distribution range of *Sinopotamon* contracted during glacial periods and was restricted to multiple refugia. The current distribution range of *Sinopotamon* probably resulted from post-glacial expansion along river drainages from independent refuges. The distribution of *Sinopotamon* extending to Sichuan basin was most likely derived from the refuge in southeastern Daba Mountains. For example, *S. acutum* and *S. davidi*, whose present distribution is ranged across Sichuan basin, were likely derived from the southeast of the Daba Mountains. Species in the north of the middle reach of the Yangtze River, and middle and lower reaches of the Huaihe River were possibly derived from the central refuge. For example, *S. honanese* probably expanded from the central refuge. *S. yangtsekiense* includes three subspecies, *S. y. yangtsekiense*, *S. y. shanxianense* and *S. y. tongbaiense*. The model niche of the LGM for *S. yangtsekiense* was located in the central and southeastern refuges. We presumed that *S. yangtsekiense* could expand northwest and northeast respectively from the two separate refuges after glaciations, and their local adaptation generated phenotypic discontinuities of the male first pleopod among geographical populations [121]. Extant species found south of the middle reaches and in the lower reaches of the Yangtze River (including the Jiangnan Hills and coastal provinces) probably expanded from the southeastern refuge. For example, *S. lansi* and *S. fukienense* probably expanded from the southeastern refuge. Such multiple separate refuges resemble that of related organisms, such as the black spotted frog, whose extant populations are derived from two isolated glacial refuges located in eastern and southwestern China [32]. In addition, several freshwater fishes were revealed to retreat to southern waters during glacial periods, some remained in their suitable southern habitat during post-glaciations [116], [117], [118], and others recolonized north [119]. The development of numerous tributaries of the Yangtze River and the highly heterogeneous topography of mountains provided ideal conditions for dispersal and differentiation of the *Sinopotamon*. Moreover, the occurrence of multiple glacial refuges could further promote inter- and intraspecific differentiation within the genus. This idea is supported by the high species diversity and areas of endemism obtained in our study (Figs. 3 & 4c). The patterns observed in *Sinopotamon* may be indicative of what took place generally amongst amphibious organisms adapted to warm temperate and subtropical regions in central eastern China during glacial and post-glacial cycles.

Further evidence using phylogeographical analyses based on molecular data are needed to help understand the historical processes that have shaped current distribution patterns of *Sinopotamon* and verify the location of multiple glacial refuges.

## Supporting Information

Figure S1 **Predicted distributions of** ***S. acutum*** **,** ***S. davidi*** **and** ***S. depressum*** **generated in Maxent. A.** ENMs for current condition. **B.** ENMs for the Last Glacial Maximun.
(TIF)

Figure S2 **Predicted distributions of** ***S. honanese, S. shensiense*** **and** ***S. yangtsekiense*** **generated in Maxent. A.** ENMs for current condition. **B.** ENMs for the Last Glacial Maximun.
(TIF)

Figure S3 **Predicted distributions of** ***S. lansi, S. chekiangense*** **and** ***S. fukienense*** **generated in Maxent.** **A.** ENMs for current condition. **B.** ENMs for the Last Glacial Maximun.
(TIF)

Table S1 **The heuristic estimate of relative contributions of the environmental variables to the Maxent model.**
(DOC)

Table S2 **The species diversity of** ***Sinopotamon*** **in each OGU.**
(DOC)

Table S3 **The endemic** ***Sinopotamon*** **species used to delimit AOEs.**
(DOC)
