# Supplementary figures and images for: Patterns of Diversity, Areas of Endemism, and Multiple Glacial Refuges for Freshwater Crabs of the Genus Sinopotamon in China (Decapoda: Brachyura: Potamidae)
Source: PLoS One. 2013 Jan 4;8(1):e53143. doi: 10.1371/journal.pone.0053143 (PMC3537761; doi:10.1371/journal.pone.0053143)

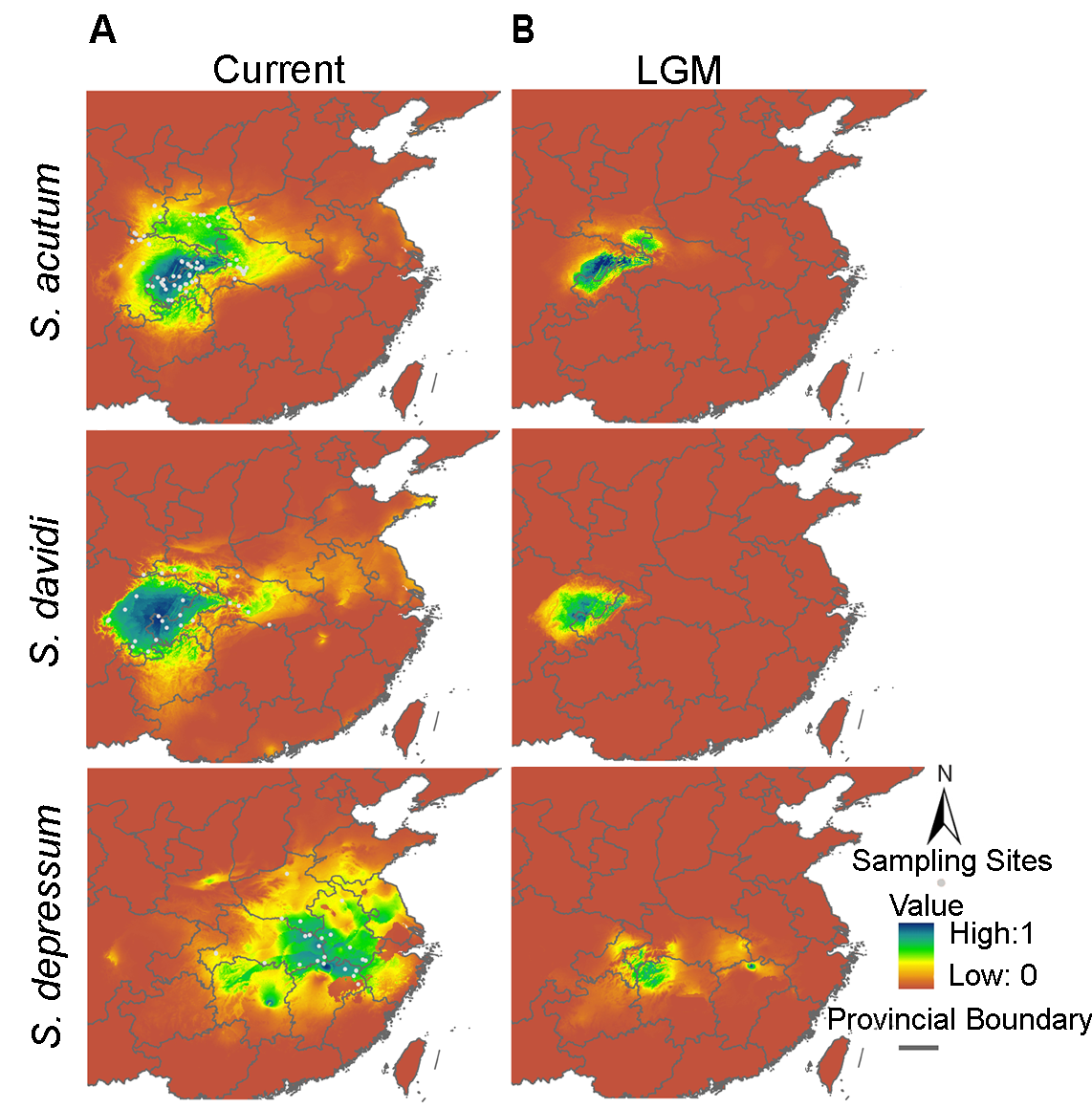

Supplement: Figure S1 — Predicted distributions of S. acutum , S. davidi and S. depressum generated in Maxent. A. ENMs for current condition. B. ENMs for the Last Glacial Maximun. (TIF) [file pone.0053143.s001.tif]

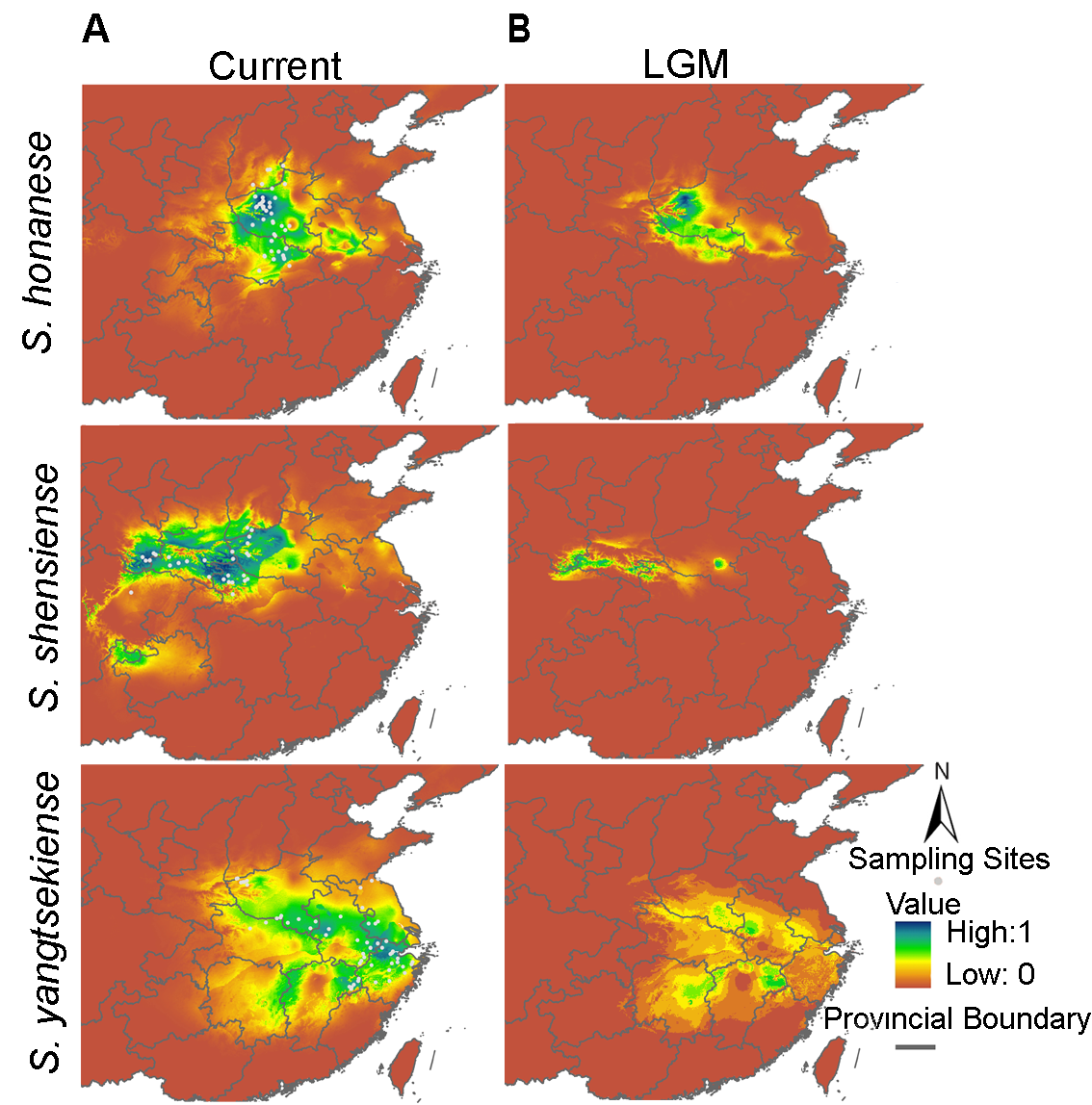

Supplement: Figure S2 — Predicted distributions of S. honanese, S. shensiense and S. yangtsekiense generated in Maxent. A. ENMs for current condition. B. ENMs for the Last Glacial Maximun. (TIF) [file pone.0053143.s002.tif]

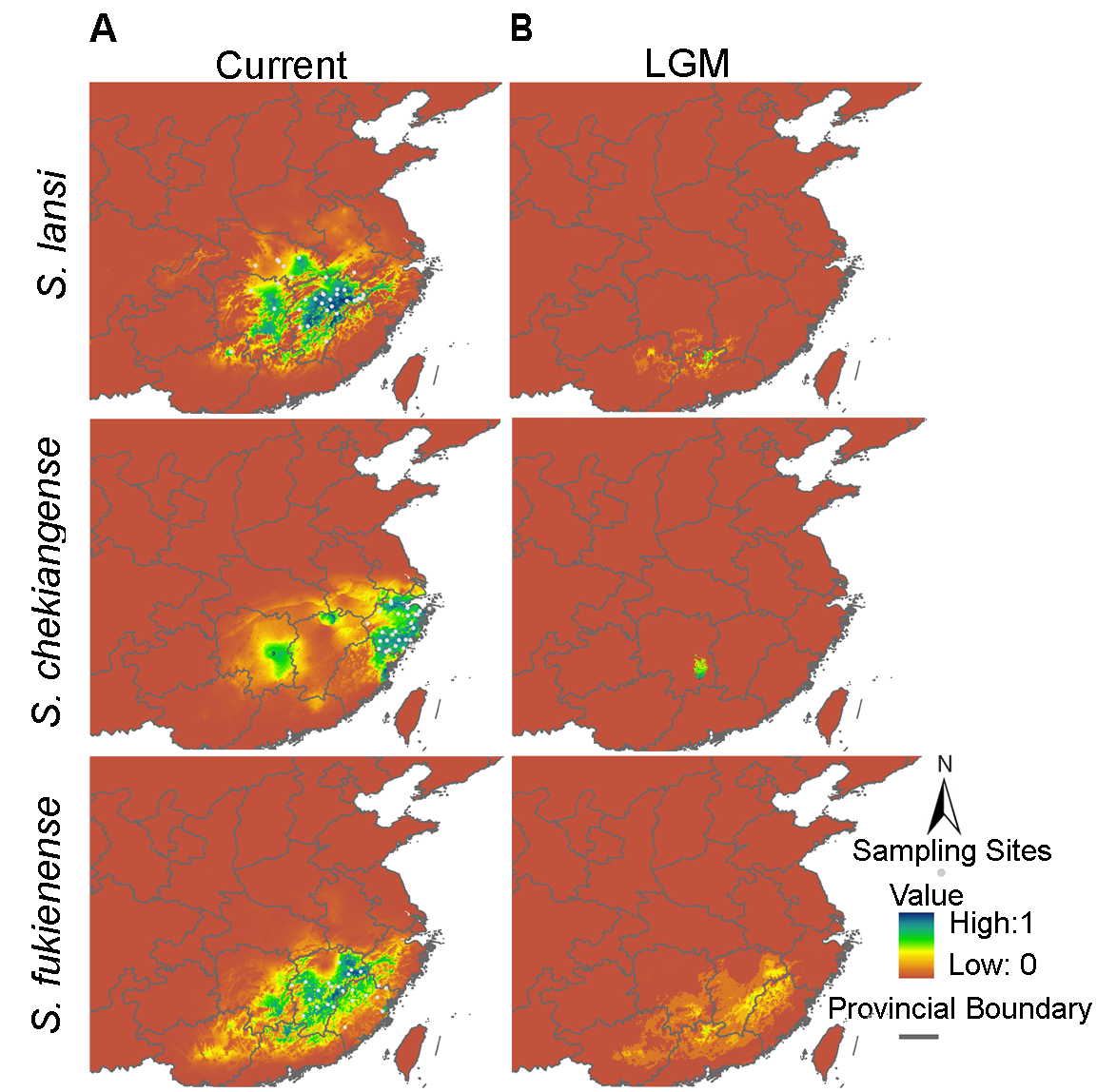

Supplement: Figure S3 — Predicted distributions of S. lansi, S. chekiangense and S. fukienense generated in Maxent. A. ENMs for current condition. B. ENMs for the Last Glacial Maximun. (TIF) [file pone.0053143.s003.tif]
