# Supplementary material for: Patterns of Diversity, Areas of Endemism, and Multiple Glacial Refuges for Freshwater Crabs of the Genus Sinopotamon in China (Decapoda: Brachyura: Potamidae)
Source: PLoS One. 2013 Jan 4;8(1):e53143. doi: 10.1371/journal.pone.0053143 (PMC3537761; doi:10.1371/journal.pone.0053143)
Supplement: Table S1 — The heuristic estimate of relative contributions of the environmental variables to the Maxent model. (DOC) [file pone.0053143.s004.doc]

Table S1 The selected environmental variables for genus and single species and the variables’ relative contributions to the Maxent model.

| Species | *S.* spp | *S. acutum* | *S. chekiangense* | *S. lansi* | *S. honanese* | *S. yangtsekiense* | *S. davidi* | *S. depressum* | *S. shensiense* | *S. fukienense* |
| --- | --- | --- | --- | --- | --- | --- | --- | --- | --- | --- |
| SSelected variables  (Contributions %) | BIO6 (64.9) | BIO12 (24.5) | BIO19 (74.9) | BIO12 (36.4) | BIO12 (31.6) | BIO12 (30.1) | BIO2 (29.8) | BIO12 (37.8) | BIO6 (35.6) | BIO19 (85.6) |
|  | BIO3 (11.6) | BIO2 (22.7) | BIO15 (7.8) | BIO5 (35.7) | BIO9 (26.9) | BIO7 (23.1) | BIO12 (28.1) | BIO6 (25.2) | BIO12 (23.4) | BIO5 (10.6) |
|  | BIO7 (7.3) | BIO19 (14.5) | BIO17 (6.1) | BIO6 (9.3) | BIO7 (19.1) | BIO6 (19) | BIO14 (15) | BIO7 (23.8) | BIO3 (13.4) | BIO3 (3) |
|  | BIO15 (4.5) | BIO3 (10.1) | BIO8 (3.4) | BIO2 (7.5) | BIO5 (11.9) | BIO15 (16.2) | BIO6 (14) | BIO18 (5) | BIO14 (12.6) | BIO15 (0.4) |
|  | BIO12 (4.2) | BIO6 (9.3) | BIO1 (2.6) | BIO4 (4.7) | BIO13 (9.8) | BIO3 (5.4) | BIO3 (7) | BIO5 (3.2) | BIO15 (6.4) | BIO14 (0.3) |
|  | BIO5 (2.9) | BIO15 (8) | BIO6 (1.2) | BIO7 (4) | BIO8 (0.8) | BIO8 (2.3) | BIO8 (4.5) | BIO9 (2.9) | BIO7 (4.4) | BIO6 (0.1) |
|  | BIO8 (1.1) | BIO7 (5.7) | BIO14 (1.1) | BIO9 (1) |  | BIO9 (2.2) | BIO7 (1.4) | BIO15 (1.9) | BIO8 (2.9) |  |
|  | BIO19 (1) | BIO17 (3.3) | BIO13 (1) | BIO15 (0.6) |  | BIO18 (1.2) | BIO15 (0.1) | BIO8 (0.2) | BIO13 (1.5) |  |
|  | BIO13 (0.9) | BIO5 (1.5) | BIO4 (0.6) | BIO8 (0.5) |  | BIO13 (0.3) |  |  |  |  |
|  | BIO2 (0.8) | BIO8 (0.4) | BIO12 (0.5) | BIO18 (0.3) |  | BIO5 (0.2) |  |  |  |  |
|  | BIO18 (0.7) | BIO18 (0.1) | BIO9 (0.3) |  |  |  |  |  |  |  |
|  | BIO9 (0.2) |  | BIO3 (0.2) |  |  |  |  |  |  |  |
|  |  |  | BIO10 (0.2) |  |  |  |  |  |  |  |
