# Supplementary material for: Patterns of Diversity, Areas of Endemism, and Multiple Glacial Refuges for Freshwater Crabs of the Genus Sinopotamon in China (Decapoda: Brachyura: Potamidae)
Source: PLoS One. 2013 Jan 4;8(1):e53143. doi: 10.1371/journal.pone.0053143 (PMC3537761; doi:10.1371/journal.pone.0053143)
Supplement: Table S2 — The species diversity of Sinopotamon in each OGU. (DOC) [file pone.0053143.s005.doc]

Table S2 The species diversity of *Sinopotamon* in each OGU.

| OGUs | Species diversity |
| --- | --- |
| E9, F2, F4, F8, F9, G5, G8, H7 | 1 |
| A2, A4, A5, A6, A7, B10, D3, F6, G6 | 2 |
| B3, B9, C3, C5, D10, E2, E3, E4, E8 | 3 |
| C10, D6, D9, E6, E7 | 4 |
| A8, B7, D1, F5 | 5 |
| C1, D2, D7, D8 | 6 |
| B8, C2, E5 | 7 |
| B4, B5, B6, C6, C7 | 9 |
| C4, C9 | 10 |
| C8, D4 | 11 |
| D5 | 15 |
