# Supplementary material for: Patterns of Diversity, Areas of Endemism, and Multiple Glacial Refuges for Freshwater Crabs of the Genus Sinopotamon in China (Decapoda: Brachyura: Potamidae)
Source: PLoS One. 2013 Jan 4;8(1):e53143. doi: 10.1371/journal.pone.0053143 (PMC3537761; doi:10.1371/journal.pone.0053143)
Supplement: Table S3 — The endemic Sinopotamon species used to delimit AOEs. (DOC) [file pone.0053143.s006.doc]

Table S3 The endemic Sinopotamon species used to delimit AOEs.

| AOEs | Species |
| --- | --- |
| Northeastern edge of the Yunnan-Guizhou Plateau (AOE 1)  Eastern Funiu Mountains  (AOE 2)  The Jiangnan Hills (AOE 3) | *Sinopotamon teritisum* Dai *et al*., 1986  *Sinopotamon* *davidi davidi* Rathbun, 1904  *Sinopotamon acutum* Dai, 1997  *Sinopotamon* *nanum* Dai & Chen, 1990  *Sinopotamon quadratapodum* Dai, Chen, Zhang & Lin, 1986  *Sinopotamon* *depressum depressum* Dai & Fan, 1979  *Sinopotamon yangtsekiense yangtsekiense* Bott, 1967  *Sinopotamon* *shensiense* Rathbun, 1904  *Sinopotamon* *styxum* Dai, 1990  *Sinopotamon* *denticulatum* Milne-Edwards, 1853  *Sinopotamon* *exiguum* Dai, 1997  *Sinopotamon* *convexum* Dai, 1995  *Sinopotamon* *xiangxiense* Dai, 1995  *Sinopotamon cladopodum* Dai *et al*., 1986  *Sinopotamon* *yueyangense* Dai, 1995  *Sinopotamon* *lansi* Doflein, 1902  *Sinopotamon* *honanense* Dai *et al*., 1975  *Sinopotamon depressum shangchengense* Dai, 1999  *Sinopotamon* *yangtsekiense yangtsekiense* Bott, 1967  *Sinopotamon yangtsekiense tongbaiense* Dai & Chen, 1981  *Sinopotamon* *planum* Dai, 1992  *Sinopotamon depressum depressum* Dai & Fan, 1979  *Sinopotamon wanzaiense* Dai, Zhou & Peng, 1995  *Sinopotamon styxum* Dai, 1990  *Sinopotamon lansi* Doflein, 1902  *Sinopotamon yixianense* Du *et al*., 1981  *Sinopotamon uneaquum* Dai & Jiang, 1991  *Sinopotamon bilobatum* Dai & Jiang, 1991  *Sinopotamon fukienense* Dai & Chen, 1979  *Sinopotamon siguqiaoense* Dai, Zhou & Peng, 1995  *Sinopotamon jiujiangense* Dai, Zhou & Peng, 1995  *Sinopotamon yangtsekiense yangtsekiense* Bott, 1967  *Sinopotamon jianglense* Dai, 1993  *Sinopotamon jichiense* Du *et al*., 1978 |
